# Nurses’ knowledge in ethics and their perceptions regarding continuing ethics education: a cross-sectional survey among nurses at three referral hospitals in Uganda

**DOI:** 10.1186/s13104-015-1294-6

**Published:** 2015-07-29

**Authors:** Charles Peter Osingada, Gorrette Nalwadda, Tom Ngabirano, John Wakida, Nelson Sewankambo, Damalie Nakanjako

**Affiliations:** Department of Internal Medicine, School of Medicine, Makerere College of Health Sciences, Kampala, Uganda; Department of Nursing, School of Health Sciences, Makerere College of Health Sciences, Kampala, Uganda; Ministry of Health, Uganda Nurses and Midwife’s Council, Kampala, Uganda; Infectious Diseases Institute, Makerere University College of Health Sciences, Kampala, Uganda

**Keywords:** Nursing ethics, Knowledge, Perceptions, Attitudes, Continuing nursing education

## Abstract

**Background:**

High disease burden and scarcity of healthcare resources present complex ethical dilemmas for nurses working in developing countries. We assessed nurses’ knowledge in ethics and their perceptions about Continuous Nurses’ Ethics Education (CNEE) for in-service nurses.

**Methods:**

Using an anonymous, pre-tested self-administered questionnaire, we assessed nurses’ knowledge in basic ethics concepts at three regional hospitals in Uganda. Adequate knowledge was measured by a score ≥50% in the knowledge assessment test. Nurses’ perceptions on CNEE were assessed using a six-point Likert scale.

**Results:**

Of 114 nurses, 91% were female; with mean age 44.7 (SD 10) years. Half were diploma, 47 (41%) certificates, 6 (5%) bachelors’ degrees and one masters’ level training. Overall, 18 (16%) scored ≥50% in the ethics knowledge test. Nurses with diploma or higher level of nursing training were less likely to fail the ethics knowledge than certificate-level nurses (OR 0.14, 95% CI: 0.02–0.7). Only 45% had ever attended at least one CNEE session and up to 93% agreed that CNEE is required to improve nurses’ ethics knowledge and practice.

**Conclusions:**

Nurses exhibited low knowledge in ethics and positive attitudes towards CNEE. We recommend structured CNEE programs to address basic concepts in nursing ethics and their application in clinical practice.

**Electronic supplementary material:**

The online version of this article (doi:10.1186/s13104-015-1294-6) contains supplementary material, which is available to authorized users.

## Background

High disease burden, coupled with scarcity of healthcare resources present complex ethical issues for nurses working in developing countries. Therefore, nurses should be adequately prepared to deal with the ethical challenges amidst the high workload and resource-constrained settings. Whereas ethics of nurses is critical to the quality of nursing care, little has been documented about nurses’ knowledge in ethics, their formal and in-service ethics training in developing countries including Uganda. This paper reports on the evaluation of the ethics knowledge among nurses working at regional referral hospitals in Uganda as well as their attitudes on Continuous Nurses’ Ethics Education (CNEE) for nurses throughout their medical practice. Our results are important to inform nurses’ training curricula as well as monitoring and evaluation activities about the need to re-emphasize ethics education and practice in nursing health care delivery.

### Continuous Nurses’ Ethics Education (CNEE)

Knowledge in ethics has been shown to enhance nursing practice in (a) identification of ethical questions within practical problems, (b) development of innovative solutions for prevalent problems and (c) development of sound ethical beliefs and practices [1]. For example, nurses with both professional ethics education and in-service or continuing education are confident in their moral judgments and are more likely to use ethics resources and take moral actions [2]. In addition, nursing ethics education was reported as an important antidote to the professional ill of moral distress and a critical factor in nursing retention [3]. In Uganda, nurses are trained at various levels including certificate, diploma, degree and more recently doctoral level. At each of these levels, it is not known whether the various categories receive the knowledge, skills and competence in ethics that are required for their nursing practice. Studies dealing with the question of what is actually taught or how students have achieved the set goals are very scarce globally, and more so in developing countries [4]. To date, there is limited documentation on the current knowledge and competencies in ethics among practicing nurses in Uganda. Similarly, there are no formal continuing nursing ethics education programs in the country.

### Purpose of the study and research questions

This study was designed to evaluate nurses’ ethics knowledge and practice as well as identify the nurses’ perceptions towards continuing nurses’ ethics education (CNEE). The research questions were;

1. To determine the level of ethics knowledge and associated factors, among nurses practicing at regional referral hospitals in Uganda.
2. To document the perceptions about continuous nurses’ ethics education (CNEE) among nurses practicing at regional referral hospitals in Uganda.

Our findings emphasize the need to review and improve both pre-service and in-service ethics training in Uganda. Given the importance of nursing ethics education in improving nursing practice, coupled with the increasing demands, awareness and expectations from clients, it is imperative that due attention is given to pre-service as well as in-service nursing ethics education in Uganda.

## Study design and methodology

### Study design, participants and study setting

This was a cross-sectional study of nurses (midwives, enrolled nurses, diploma nurses as well as holders of bachelor’s degrees in nursing) working in three regional referral hospitals in Uganda. There are about 51,162 nurses and midwives in Uganda providing nursing services at different levels of health delivery. The regional referral hospitals were purposively selected and all nurses on duty were given a pre-coded, self-administered questionnaire. A sample size of 384 participants was estimated using Leslie Kish formula (n = z^2^P(1 − P)/d^2^) [5]; given that there were no previous studies on nurses’ knowledge in ethics, we assumed that 50% (P = 0.1) of the nurses and midwives possessed adequate knowledge in ethics at the three regional referral hospitals (Mbale, Mbarara and Lira). The margin of error was taken as 5% and a standard z of 1.96 was used [5]. The sample was adjusted to 187 participants using the formula n = n_0_/(1 + n_0_ − 1/N). The total number of nurses and midwives in the three regional referral hospitals was 365. One hundred and twenty-one were from Mbale, 133 from Mbarara, and 111 from Lira hospital. Proportionate sampling was used to obtain the number of participants from each hospital. Thus a total of 63 participants were recruited from Mbale hospital, 68 from Mbarara hospital and 56 from Lira hospital. We excluded foreign-trained nurses/midwives and nursing assistants (health care providers without formal training). Permission to conduct the study was sought from the leadership of each of the referral hospitals. Written informed consent was sought from the study participants.

### Data collection

Nurses’ ethics knowledge was evaluated using a pre-coded, pre-tested, anonymous self-administered questionnaire which consisted of three sections: (a) demographic information, (b) an ethics knowledge test, and (c) perceptions/attitudes Likert scale (see Additional file 1). The knowledge test questions were developed by the investigators since no appropriate tool could be located from previous studies. The test comprised seven short answer questions and 32 multiple choice questions. The questions assessed participants’ knowledge of basic concepts in nursing ethics and their application in nursing care. The concepts tested included: informed consent, confidentiality, veracity, principles of ethics, ethical theories, value clarification and some aspects of Uganda Nurses and Midwives code of ethics. Items on perceptions were in a six-point Likert format, with scores ranging from 1 (strongly disagree) to 6 (strongly agree).

On the day of data collection in each of the selected hospitals, all nurses and midwives working on duty were requested to participate in the study. Participants filled the questionnaires and returned them to the investigators on the same day. The knowledge test section was marked and percentage scores of individual participants were entered into the computer for analysis.

### Data analysis

Data was double entered into Excel sheets and exported to SPSS version 17 for analysis. Participants who obtained less than 50% in the knowledge test were considered to have a low level of knowledge in nursing ethics (the 50% cutoff was chosen because it is the pass mark for our nursing training institutions). Chi square tests were performed to compare categorical variables such as gender, level of education and history of previous ethics education during nursing practice and Student’s T test was used to compare continuous variables such as age and years of practice among nurses with good knowledge of ethics (score ≥50%) and nurses with low knowledge of ethics (score <50%). A *p* value <0.05 was considered statistically significant.

### Ethical consideration

The research was carried out in compliance with the Helsinki Declaration (http://www.wma.net/en/30publications/10policies/b3/index.html). Permission to conduct the study was sought from the Makerere University School of Health Sciences Institutional Review Board (IRB) and the nurses gave informed consent to participate in the study.

## Results

### Participants’ characteristics

The findings reported below are from 114 out of 187 (61%) participants who returned the study questionnaires. A total of 114 nurses and midwives were surveyed from three regional referral hospitals. Majority of the respondents (90.6%) were female. The mean age was 44.7 (SD 10.1), and it ranged from 24 to 59 years. Slightly over half of the participants had a diploma in nursing/midwifery, 47 (41.2%) had certificates, 6 (5.3%) had bachelors’ degrees and only one participant had a masters’ degree.

### Nurses’ ethics knowledge and associated factors

Overall, 18 (15.8%) of the nurses/midwives obtained a score ≥50% in the ethics knowledge assessment test. The mean percentage score was 37.1 (SD 11.5) and it ranged from 14 to 68% (see Table 1).

**Table 1:** Social demographic characteristics of 114 nurses evaluated for ethics knowledge

Variable	Low knowledge (score ≤ 50%) n = 96	Adequate knowledge (score ≥ 50%) n = 18	OR (95% CI)	P value
Gender				
Female	87 (91%)	17 (94%)	0.6 (0.1–4.8)	0.599
Male	9 (9%)	1 (6%)	1.0	
Age				
<45	49 (51%)	6 (33%)	2.1 (0.7–6.0)	0.168
≥45	47 (49%)	12 (67%)	1.0	
Level of education				
Diploma and above	51 (53%)	16 (89%)	0.14 (0.02–0.7)	0.005*
Certificate	45 (47%)	2 (11%)	1.0	
Duration in practice				
<16 years	46 (48%)	6 (33%)	1.8 (0.6–5.3)	0.254
≥16 years	50 (52%)	12 (67%)	1.0	
Ever attended CME/CNE in ethics				
Yes	44 (46%)	7 (39%)	1.3 (0.5–3.7)	0.587
No	52 (54%)	11 (61%)	1.0	

* Significant findings p value <0.05.

Nurses who had attained a diploma or higher level of education were less likely to score below 50% in the knowledge test compared with nurses who had a certificate (OR 0.14, 95% CI: 0.02–0.7). Participants who had practiced for 16 years or more were more likely to score ≥50% in the knowledge test compared with those who had practiced for less than 16 years but the difference was not statistically significant (OR 1.8; 95% CI: 0.6–5.3).

### Nurses’ perspectives on continuous nurses’ ethics education (CNEE)

Up to 93% of the participants agreed that it is important to receive CNEE, and 109 (95.6%) agreed that they needed training in nursing ethics. The participants were also asked about acceptability of CNEE and 107 (93.9%) of them indicated that CNEE was acceptable. In addition, 74 (65%) of the nurses were dissatisfied with the quality of ethics training provided to diploma and certificate nursing students, and only 44.7% had ever attended at least one continuing education in ethics (see Table 2).

**Table 2:** Perceptions towards continuing nursing ethics education (CNEE) among nurses at three referral hospitals in Uganda

Perception/attitudes (N = 114)	Agree no (%)	Disagree no (%)
Important to conduct CNEE	106 (93%)	8 (7%)
Relevant to provide CNEE	101 (89%)	13 (11%)
CNEE will improve nurses’ accountability	106 (93%)	8 (7%)
CNEE will improve nurses’ reputation	99 (87%)	15 (13%)
CNEE will improve patient/client care	104 (91%)	10 (9%)
Perceive that ethically challenging situations are common in practice	89 (78%)	25 (22%)
Perceive themselves to be in need of training in nursing ethics	109 (96%)	5 (4%)

## Discussion

### Knowledge in nursing ethics

To our knowledge, this is the first study to assess nurses’ knowledge in ethics and their perceptions towards continuing nursing ethics education in Uganda. We found low knowledge about the basic concepts of ethics that are relevant to nursing care such as; informed consent, confidentiality, veracity, principles of ethics, ethical theories, value clarification as well as general aspects of the national nurses and midwives code of conduct. Only 15% of the respondents scored ≥50% in the ethics knowledge test. Our results on knowledge of ethics are slightly lower than reports from Germany where Eilts-Köchling and colleagues [6] showed that only 25% of nurse respondents knew about their codes of ethics. Similar reports among advanced practice nurses show that nurses exhibited low levels of knowledge in ethics despite high levels of confidence during their practice in the USA [7]. These results imply that there is a need to increase nurses’ knowledge of ethics, more so because adequate working knowledge in ethics is postulated to transform nurses’ attitudes and approaches towards provision of quality nursing care in Uganda [8].

Age, gender, and duration in practice were not associated with more knowledge in nursing ethics. These results agree with Gallagher’s views that ethical competence does not only emerge during the development of general professional competence, but rather requires specific ethics education [9]. It is however important to note that majority of the respondents were female, reflecting the predominantly female nursing profession in Uganda, so we did not make conclusive results on the association of gender and knowledge of ethics among nurses. Nurses who had attained a diploma or higher academic qualification were more likely to score above the knowledge test pass mark of 50% when compared with nurses that had certificate-level training. The authors postulate that the teaching of nursing ethics might be more robust during the diploma and degree-level nurses’ training than that at the certificate level. A study by Harrowing and Mill reported that Ugandan nurses experienced moral distress as they were haunted by their inability to ease patient suffering due to limited choices of quality health care [8]. Given the challenges facing health care delivery in resource-limited settings, nurses acknowledge the need for continuous ethics education as a strategy to transform their attitudes and approaches toward the provision of nursing care amidst the high patient load and limited facilities for health care [8].

Although outside the scope of this study, we recognize need to evaluate the various nurse training curricula and modes of teaching ethics at certificate, diploma and degree-level nursing training in order to inform targeted training interventions to increase ethics knowledge among practicing nurses. In addition, the nursing regulatory bodies could explore the implementation of in-service ethics training to ensure that nurses’ approaches and clinical decisions are in conformity with national and universal ethical standards such as those by the International Council of Nurses [10].

### Perceptions towards Continuous Nursing Ethics Education (CNEE)

Overall, the respondents had positive perceptions towards CNEE. Majority (93%) of the nurses and midwives agreed that CNEE was important to for a sustainable high standard of ethics knowledge among nurse providing health care in Uganda. Our results are consistent with reports by Harrowing and Mill where Ugandan nurses acknowledged the need for CNEE sessions to support in-service nurses to cope with the challenges facing their nursing practice [8]. Our results are similar to reports from physicians and nurses in Barbados where it was observed that 90% of the nurses reported that knowledge of ethics was important in their work as health care providers [11]. Similarly, in China, 89% of 542 nurse respondents reported that medical ethics education was necessary for all medical staff [12]. The positive attitudes towards CNEE, as shown by nurses in this study, indicate great potential for a structured CNEE program to improve ethics knowledge, skills and competences among nurses at regional referral hospitals in Uganda. However, only 45% of the respondents reported having attended at least one CNEE session during their professional nursing career. This result also implies that there is a need to rejuvenate efforts put into CNEE [7, 13]. Given that CNEE was previously acknowledged as an important source of knowledge in ethics [2, 11], and the positive attitude towards CNEE shown in this study, there is a need to increase in-service nursing ethics education in order to prepare the nurses to cope with current and future challenges to ensure quality nursing care delivery within the various resource-constrained settings in the developing world. These results inform the current development of national strategies to strengthen Continuing Professional Development (CPD) programs for nurses and midwives in the country.

It is important to note that the knowledge assessment tool used in this study was developed by the investigators based on widely known basic concepts in nursing ethics and the requirements of national nurses’ and midwifery code of conduct. The tool was pre-tested among nurses in similar settings and we encourage its validation for use in routine evaluation of ethics knowledge among nurses.

### Limitations

This study did not evaluate the nurses’ ethics training curricula at the various nursing schools. Therefore, it remains unclear whether the low ethics knowledge among nurses was a result of limited ethics training within nursing training programs in Uganda. We recommend further research to evaluate nurses’ formal and in-service ethics training in order to inform regular evaluation of the ethics curriculum as well as the effectiveness of ethics education to both nurses and patients.

## Conclusions

Nurses exhibited low knowledge in ethics. Nurses with diploma or higher level of education had more knowledge in ethics than the certificate-level nurses. All nurses exhibited positive attitudes towards continuous nursing ethics education (CNEE). We recommend development of structured CNEE programs to increase ethics knowledge and competences among in-service nurses. Further research is required in the area of ethics training for nurses to inform the monitoring and evaluation activities for ethics training and practice among nurses in Uganda and other resource-constrained settings.

## Additional file

Additional file 1: Assessment of nurses’ knowledge in ethics and their perceptions on continuing ethics education.
