# Supplementary material for: Nurses’ knowledge in ethics and their perceptions regarding continuing ethics education: a cross-sectional survey among nurses at three referral hospitals in Uganda
Source: BMC Res Notes. 2015 Jul 29;8:319. doi: 10.1186/s13104-015-1294-6 (PMC4517354; doi:10.1186/s13104-015-1294-6)
Supplement: Additional file 1: — Assessment of nurses’ knowledge in ethics and their perceptions on continuing ethics education. [file 13104_2015_1294_MOESM1_ESM.docx]

**ASSESSMENT OF NURSES’ KNOWLEDGE IN ETHICS AND THEIR PERCEPTIONS ON CONTINUING ETHICS EDUCATION**

**Appendix I: Self-administered questionnaire**

**Section A**

1. What is your professional qualification……………………………………….(e.g. Enrolled Nurse, etc )

1. What is your age ……………………. ( in completed years)
2. What is you gender ( circle one)
3. Female
4. Male
5. What is your highest academic qualification (circle one)
6. Certificate
7. Diploma
8. Bachelor’s degree
9. Masters degree
10. For how long have you practiced as a Nurse /Midwife? ………( in completed years)
11. Have you ever attended training or CME/CNE in ethics?
12. Yes
13. No

**SECTION B**

**The following section is intended to assess your current knowledge in nursing ethics. This will guide in developing a Continuous Professional Development program. Therefore it is important that you answer each question as honestly as possible and to the best of your knowledge.**

**For questions 7 to 13, write your responses in the spaces provided.**

1. What does nursing ethics mean?

________________________________________________________________________________________________________________________________________________________________________________________________________________________________________________________________________________________________________________________________________________________________________

1. List the four principles of bioethics
2. _____________________________________________________________________
3. _____________________________________________________________________
4. _____________________________________________________________________
5. _____________________________________________________________________
6. Define informed consent?

________________________________________________________________________________________________________________________________________________________________________________________________________________________________________________________________________________________________________________________________________________________________________

1. List at least two moral or ethical theories
2. ____________________________________________________________________
3. ____________________________________________________________________
4. What does the concept veracity mean?

________________________________________________________________________________________________________________________________________________

1. A nurse who discloses a patient information to a colleague in a taxi violates a patient right to__________________________________________________________________
2. The concept of etiquette refers to ________________________________________________________________________________________________________________________________________________

**For questions 14 to 45 please circle the most correct answer**

1. The defining marks of a profession are:
   1. Competence in a specialized body of knowledge and skills
   2. An acknowledgement of specific duties & responsibilities toward the individual it serves and toward society
   3. The rights to train, admit, discipline and dismiss its members for failure to sustain competence or observe the duties and responsibilities
   4. All the above
   5. None of the above.
2. Moral distress occurs when:
   1. A nurse indentifies a moral problem but is unsure of the morally correct action
   2. There are two or more mutually exclusive moral claims and both seem to have equal weight
   3. The nurse knows the morally correct action and feels responsibility to the patient, but institutional or other restrains make it nearly impossible to follow through the appropriate action
   4. A nurse is uncomfortable with the situation, but cannot figure out the problem
3. The following are examples of unethical behavior except:
   1. Disclosure of information regarding a patient without patient’s/ client’s permission
   2. Deliberate failure to obtain consent from a patient/client
   3. Intentionally administering a lethal chemical to a patient
   4. Failing to update one’s Continuing Professional Development
4. This corresponds to the personal belief of a client regarding a given idea, custom, or attitude that influences her behavior and decisions:
   1. Ethics
   2. Morals
   3. Values
   4. Bioethics
5. A patient is diagnosed with a terminal cancer; his family asked his nurse to explain to him his illness on their behalf. What principle guides this situation?
   1. Autonomy
   2. Veracity
   3. Beneficence
   4. The principle of double effect
6. A nurse who accepts to participate in a television commercial while wearing her uniform violates;
   1. Ugandan law
   2. Physician code of conduct
   3. Professional code of conduct and ethics for nurses and midwives in Uganda
   4. None of the above
7. The following statements below are acts of nursing negligence, except
   1. Use of defective equipment
   2. Disagreeing with the doctor
   3. Careless execution of doctor’s order
   4. Errors in administering medication
8. According to the Uganda professional code of conduct and ethics for nurses and midwives, nurses shall not:
   1. Engage in activities that bring discredit to his or her profession or to the delivery of health services
   2. Perform his/her duties under the influence of alcohol
   3. Support or become associated with occult or unscientific practices professing to contribute to health care.
   4. All the above
   5. None of the above
9. An informed consent is an agreement that allows a certain procedure to happen. Which is true about it?
   1. Jesica is being given the relevant information of her chemotherapy
   2. Opio, 18 years old who refuses a treatment is allowed to sign his waiver in the absence of his guardian.
   3. Wendo, mother of baby Timothy, signed the consent form for his blood transfusion.
   4. All of the above
10. You are one of the vaccinators in the measles campaign. One mother hesitates since she verbalized that it would hurt her son. As a well- versed nurse about ethical considerations you explained to the mother that it may cause a discomfort but it will protect her son from acquiring measles. Which of these did you utilize:
    1. Nonmaleficence
    2. Justice
    3. Beneficence
    4. Autonomy
11. According to the preamble of the professional code of conduct and ethics for Uganda nurses and midwives, nurses and midwives are Registered or Enrolled to:
    1. Promote health
    2. Prevent illness
    3. Restore health
    4. Alleviate suffering
    5. All of the above
12. Standards we use to determine right from wrong, or good from bad behavior are known as:
13. Beliefs
14. Ethics
15. Values
16. Attitudes
17. The fundamental responsibility of a nurse according to the International Council of Nurses code for nurses are the following apart from
18. Prevention of illness
19. Promotion of health
20. Restoration of health
21. Aggravation of suffering
22. To make autonomous decisions and actions, clients must be offered enough information and must be:
23. Free of internal and external influences
24. A minor
25. Unconscious
26. Comatose patient
27. Informed consent is a process that promotes
28. Autonomy
29. Nonmaleficence
30. Beneficence
31. Justice
32. Free consent is a willingness to participate in a situation related to informed consent. There are constraints related to voluntariness, which are internal and external. Internal constraints include which of the following.
33. Coercion
34. Fraud
35. Undue violence
36. Significant trauma
37. A wrong dose of drug is administered by the nurse. The nurse is responsible to whom?
38. Client
39. Physician who ordered the drug
40. Society
41. All of the above
42. Being answerable for one’s own action is assuming:
43. Accountability
44. Responsibility
45. Nonmaleficence
46. Veracity
47. Before the nurse administered the client’s medication she assessed the client’s needs for the drug, and followed the “rights” in drug preparation and administration. After the nurse has given the dose, she evaluated the client’s response to the medication given. The nurse is promoting:
48. Accountability
49. Autonomy
50. Responsibility
51. Veracity
52. The personal conviction that something is absolutely right or wrong in all situations is called:
53. Values
54. Morals
55. Ethics
56. Standards of practice
57. The principles or standards that influence behavior and decisions making based on experience, religion, education and culture are called:
58. Values
59. Morals
60. Ethics
61. Standards of practices
62. Nurse’s code of ethics is a set of ethical principles generally accepted by members of the profession. The standards or principles that a nurse must observe in the practice should promote which duties.
63. Use judgment in relation to individual competence when accepting and delegating responsibilities.
64. Plays a major role in determining and implementation of desired standards of nursing practice
65. Active in developing a core of professional knowledge
66. Acts through professional organization & participates in establishing and maintaining equitable social and economic working conditions
67. The duty to respect privileged information is called
68. Fidelity
69. Justice
70. Veracity
71. Confidentiality
72. Professional accountability serves for the following purpose except:
73. To provide basis for ethical decision
74. To respect the decision of the client
75. To maintain standards of health
76. To evaluate new professional practice and re-assess existing ones
77. External constraints can influence voluntariness in situations related to informed consent. These constraints least likely include
78. Coercion
79. Fraud
80. Undue violence
81. Significant trauma
82. When does a moral issue become an ethical issue:
83. When the choices are clearly denoting which is right and wrong
84. When valves of a person develops and changes overtime
85. When there is no acceptable reason for the wrong choice once it is done
86. When the choice is no longer clear between right and wrong
87. The execution of duties associated with nurses’ particular role is called
88. Accountability
89. Responsibility
90. Nonmaleficence
91. Veracity
92. Nurse X avoids deliberate harm, risk of harm that occurs during his performance of nursing actions. The nurse is promoting which of the following
93. Autonomy
94. Nonmaleficence
95. Beneficence
96. Justice
97. The one that promotes the philosophical and theological study of morality, moral judgments and problems is called:
98. Value
99. Morals
100. Ethics
101. Standards
102. The nurse in a unit is caring for several patients. To distribute nursing care the nurse utilizes the principle of triage due to the limited availability of resources. The nurse is promoting which of the following:
103. Fidelity
104. Justice
105. Veracity
106. Confidentiality
107. The nurse’s obligation to the client will least include
108. Consider the dignity of clients
109. Retains a commitment of welfare of the client
110. Work towards security and maintaining conditions of employment that satisfy the goals of nursing
111. Hold confidential all information about a client learned in a health care setting
112. Nursing ethics provides the standards for professional behavior. This set of standards states the duties and obligations of nurses to:
113. Clients
114. Other health professionals
115. Community
116. All of the above

**SECTION C**

**The following section is intended to assess your perceptions regarding continuing nursing ethics education. It is NOT intended to find out how much you know but rather seeks you views or general feelings or personal opinion with regard to continuing nursing ethics education for the already qualified nurses and midwives. Please note the instructions for each question and provide the appropriate response using the options given.**

**The following questions are designed to determine the way in which different people view important issues related to continuing nursing ethics education. Each item is a belief statement with which you may agree or disagree. Below each statement is a scale which ranges from strongly disagree (1) to strongly agree (6). For each item, we would like you to circle/mention the extent to which you disagree or agree with the statement. For the rest of the questions, respond to the question using the guiding stem.**

1. It is important to conduct continuing nursing ethics education for the already working nurses and midwives in Uganda.

1= Strongly disagree

2= Disagree

3= Slightly disagree

4= Slightly Agree

5= Agree

6= Strongly agree

1. It is relevant to provide continuing nursing ethics education to nurses and midwives in Uganda.

1 = Strongly disagree

2 = Disagree

3 = Slightly disagree

4 = Slightly Agree

5 = Agree

6 = Strongly agree

1. Providing continuing nursing ethics education in Uganda will improve nurses and midwifery professional accountability

1 = Strongly disagree

2 = Disagree

3 = Slightly disagree

4 = Slightly Agree

5 = Agree

6 = Strongly agree

1. Providing continuing nursing ethics education in Uganda will improve nurses reputation

1 = Strongly disagree

2 = Disagree

3 = Slightly disagree

4 = Slightly Agree

5 = Agree

6 = Strongly agree

1. Providing continuing nursing ethics education in Uganda will improve nursing care provided to patient and client.

1 = Strongly disagree

2 = Disagree

3 = Slightly disagree

4 = Slightly Agree

5 = Agree

6 = Strongly agree

1. I am encountering situations in my practice which ethically challenge me

1 = Strongly disagree

2 = Disagree

3 = Slightly disagree

4 = Slightly Agree

5 = Agree

6 = Strongly agree

1. I am in need of continuous nursing ethics training

1 = Strongly disagree

2 = Disagree

3 = Slightly disagree

4 = Slightly Agree

5 = Agree

6 = Strongly agree

1. How appropriate is continuing nursing ethics education in addressing ethical challenges faced by nurses and midwives in Uganda? Level of Appropriateness

1 = Absolutely inappropriate

2 = Inappropriate

3 = Slightly inappropriate

4 = Slightly appropriate

5 = Appropriate

6 = Absolutely appropriate

1. How important is continuing nursing ethics education in addressing ethical challenges faced by nurses and midwives in Uganda? Level of Importance

1 = Not at all important

2 = Low importance

3 = Slightly important

4 = Moderately important

5 = Very important

6 = Extremely important

1. How frequent do you experience ethically challenging situations in your work place?

Frequency

1 = Never

2 = Rarely

3 = Occasionally

4 = A moderate amount

5 = A great deal

1. How desirable is continuing nursing ethics education in addressing ethical challenges faced by nurses and midwives in Uganda? Level of Desirability

1 = Very undesirable

2 = Undesirable

3 = Neutral

4 = Desirable

5 = Very desirable

1. Considering everything, how acceptable is continuing nursing ethics education training for the already practicing nurses and midwives? Level of Acceptability

1 = Totally unacceptable

2 = Unacceptable

3 = Slightly unacceptable

4 = Slightly acceptable

5= Acceptable

6= Perfectly Acceptable

1. How satisfied are you with the nursing ethics education provided to the diploma and certificate nursing and midwifery students? Level of Satisfaction with the teaching.

1 = Completely dissatisfied

2 = Mostly dissatisfied

3 = Somewhat dissatisfied

4 = Somewhat satisfied

5= Mostly satisfied

6 = Completely satisfied

15) On a scale of 1 to 10 where 1 = no priority and 10 =high priority , circle the number that corresponds to the extent to which you generally feel that continuing nursing ethics education is a priority for practicing nurses and midwives in Uganda

Not a priority 1 2 3 4 5 6 7 8 9 10 High priority

**THANK YOU VERY MUCH FOR PARTICIPATING IN THIS ASSESSMENT**
